# Deformation of Red Blood Cells, Air Bubbles, and Droplets in Microfluidic Devices: Flow Visualizations and Measurements

**DOI:** 10.3390/mi9040151

**Published:** 2018-03-27

**Authors:** David Bento, Raquel O. Rodrigues, Vera Faustino, Diana Pinho, Carla S. Fernandes, Ana I. Pereira, Valdemar Garcia, João M. Miranda, Rui Lima

**Affiliations:** 1 Instituto Politécnico de Bragança, ESTiG/IPB, C. Sta. Apolónia, 5301-857 Bragança, Portugal; davidbento@ipb.pt (D.B); raquel.rodrigues@ipb.pt (R.O.R.); diana@ipb.pt (D.P.); cveiga@ipb.pt (C.S.F.); apereira@ipb.pt (A.I.P.); valdemar@ipb.pt (V.G.); 2 CEFT, Faculdade de Engenharia da Universidade do Porto (FEUP) Rua Roberto Frias, 4800-058 Porto, Portugal; 3 LCM—Laboratory of Catalysis and Materials—Associate Laboratory LSRE/LCM, Faculdade de Engenharia da Universidade do Porto (FEUP) Rua Roberto Frias, 4800-058 Porto, Portugal; 4 MEMS-UMinho Research Unit, Universidade do Minho, DEI, Campus de Azurém, 4800-058 Guimarães, Portugal; vera_f_87@hotmail.com; 5 Centro de Investigação em Digitalização e Robótica Inteligente (CeDRI), Instituto Politécnico de Bragança, Campus de Santa Apolónia, 5300-253 Bragança, Portugal; 6 Algoritmi R&D Centre, Campus de Gualtar, University of Minho, 4710-057 Braga, Portugal; 7 MEtRiCS, Mechanical Engineering Department, Campus de Azurém, University of Minho, 4800-058 Guimarães, Portugal

**Keywords:** red blood cells, deformation index, microfluidic devices, air bubbles, droplets, blood flow

## Abstract

Techniques, such as micropipette aspiration and optical tweezers, are widely used to measure cell mechanical properties, but are generally labor-intensive and time-consuming, typically involving a difficult process of manipulation. In the past two decades, a large number of microfluidic devices have been developed due to the advantages they offer over other techniques, including transparency for direct optical access, lower cost, reduced space and labor, precise control, and easy manipulation of a small volume of blood samples. This review presents recent advances in the development of microfluidic devices to evaluate the mechanical response of individual red blood cells (RBCs) and microbubbles flowing in constriction microchannels. Visualizations and measurements of the deformation of RBCs flowing through hyperbolic, smooth, and sudden-contraction microchannels were evaluated and compared. In particular, we show the potential of using hyperbolic-shaped microchannels to precisely control and assess small changes in RBC deformability in both physiological and pathological situations. Moreover, deformations of air microbubbles and droplets flowing through a microfluidic constriction were also compared with RBCs deformability.

## 1. Introduction

Blood flow behavior in microcirculation is strongly influenced by red blood cell (RBC) deformability as they occupy almost half of whole blood volume. When RBCs are subjected to large external flow forces, they elongate without rupture and tend to return to their original shape when the external forces are removed. Some major determinants of RBC deformability include external flow forces, cell geometry, cell internal viscosity, and membrane viscoelastic properties [1]. RBC-related diseases, such as malaria, sickle cell disease, and diabetes, can also promote significant alteration in the RBC deformability. Ever since the deformability of RBCs became a potential biomarker for several blood diseases, various experimental techniques have been developed to measure the deformation of blood cells (see Table 1). There have been several reviews discussing different techniques for measuring RBC deformability under a variety of experimental and diseased conditions [1,2,3,4]. The recent progress in microfabrication and high-speed microvisualization technology made it possible to produce microfluidic devices able to directly visualize and characterize the mechanical properties of individual cells flowing through constriction microchannels [5,6]. However, there are still few reviews focusing on the use of these kinds of microfluidic devices to measure cell deformability. Most of the recent reviews, performed by Zheng et al. [7], Tomaiuolo [8], and Xue et al. [9], have focused on single-cell devices, cylindrical glass capillaries, and in microdevices, where the shear effect is dominant. Due to the growing interest of combining the shear and extensional effect to perform deformability measurements, this review focuses on the most recent findings performed by our research group related to the deformation of RBCs flowing through hyperbolic, smooth, and sudden-contraction microchannels. Moreover, deformations of air microbubbles flowing within in vitro blood microfluidic devices are also measured and compared with RBC deformability.

## 2. Deformation of RBCs in Microfluidic Devices 

Most of the proposed microfluidic devices to perform RBC deformability characterization focus on the shear effect. Some examples from the literature are the measurements of the RBCs’ deformation under a transient high shear stress in a sudden-contraction microchannel [25] and the RBCs’ deformability through a microfluidic device with a microchannel diameter comparable to RBC size [26]. In addition to the shear effect, the extensional effect and the combination of both can be encountered in the human body, e.g., in microstenosis, in microvascular networks composed of small, irregular vessel segments, in pulmonary microvessels, and in medical instrumentation, such as the flow through syringes and syringe needles. Hence, it is important to understand the RBC mechanical properties under both shear and extensional effect.

**Figure 1:**
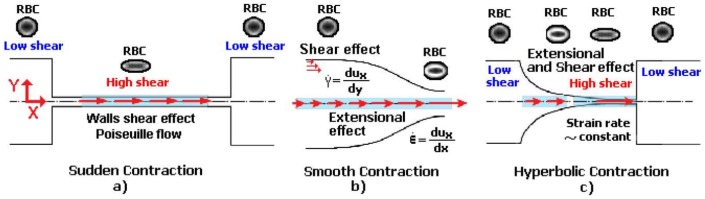
Blood flow and RBC deformability in microfluidic contractions at different geometries: (**a**) sudden contraction; (**b**) smooth contraction; and (**c**) hyperbolic contraction, adapted from [38].

Flows of blood cells through microfluidic contractions generate complex flow phenomena despite their simple geometry. The flow involves a reduction in the cross-sectional area, which generates strongly-converging flows as the fluid goes through the contraction and the blood cells exhibit a variety of shapes, such as circular, ellipse, and parachute, which depend on the rheological properties of the fluid, geometric configuration, and dimensions of the contraction. The schematic illustration in Figure 1 shows the fluid flow behavior in different kinds of microfluidic constriction channels through which the RBCs travel. In general, the flow exhibits mixed kinematics with strong extensional flow (the fluid accelerates as it is going through the contraction) along the centerline and shear flow close to the walls. The major advantage of microfluidic hyperbolic-shaped contraction (Figure 1c) is the ability to impose a constant strain rate along the centerline of the contraction, as well as to achieve high extensional and shear flows. Relevant works in the context of blood flow and RBC deformability are those performed by Sousa et al. [27], Lee at al. [28], Yaginuma et al. [29], Rodrigues et al. [30,31,32], Faustino et al. [33], Pinho et al. [19], and Calejo et al. [34], who studied the effects of the extensional flow in hyperbolic converging microchannels using blood analog fluids and in vitro blood containing different kinds of blood cells. Other works have used cross-slot microfluidic devices to investigate the deformability of different kinds of cells under the application of extensional flows [35,36,37].

RBCs flowing through microfluidic contractions are, most of the time, subjected to high shear and extensional effects and, as a result, they tend to elongate into ellipsoid shapes with their major axis aligned to the flow direction. When the cells leave the constriction region, the fluid shear forces created by the wall are removed and, consequently, RBCs tend to return to their normal resting biconcave disc shape. The deformation under controlled flow conditions provides an efficient method to generate cellular-scale mechanical stimuli. Hence, microfluidic constrictions due to the ability of performing precise control and manipulation of a small volume of samples have been gaining increasing interest to measure the deformability of RBCs for clinical proposes [5,7,8,19,28,29,30,31,32,33]. 

**Figure 2:**
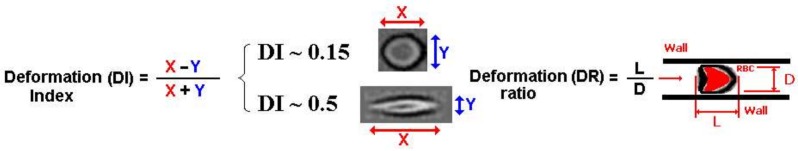
Schematic diagram of the deformation index (DI) and deformation ratio (DR) definition, adapted from [17].

The classical method to quantify the degree of deformability is by using an ellipse-fitting program. The deformation index (DI), also known as elongation index, most of the times is calculated by (X − Y)/(X + Y) where X and Y represent the major and minor lengths of the ellipse, respectively (see Figure 2). However, in microchannel capillaries, where the dimensions of the channels are smaller than the diameters of the cells, the degree of deformability should be measured as the ratio between the length of the major axis and the length of the minor axis as the RBC tends to deform in a parachute shape, as shown in Figure 2 [20,39]. This latter definition is designated as the deformation ratio (DR).

### 2.1. Deformation of RBCs in Hyperbolic Contractions 

**Figure 3:**
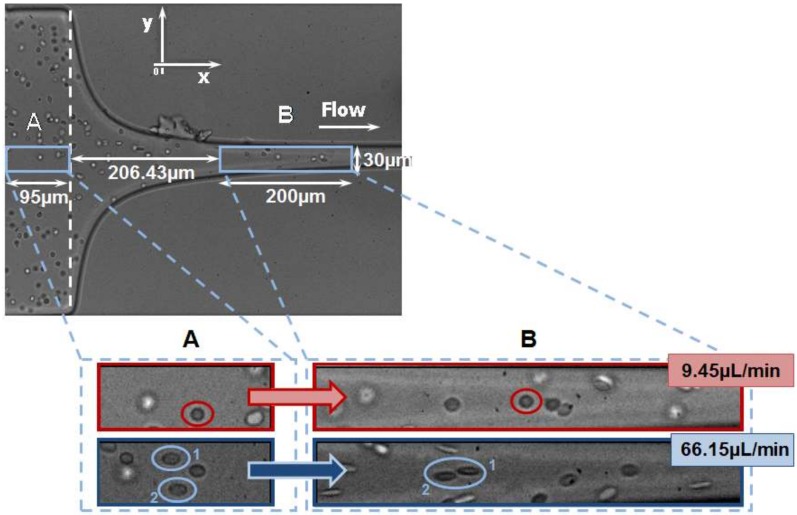
RBC deformability in a hyperbolic converging microchannel at two different regions (**A**) and (**B**) and flow rates (9.45 µL/min and 66.15 µL/min) (adapted from [40]).

Since RBC deformability became a potential clinical biomarker, several single-cell microfluidic methodologies have been developed to perform flow measurements on RBCs [6,7,8,9]. The majority of the methods to measure the RBCs’ deformability have focused on the response of the cells under simple shear flow. However, it is well known that extensional flow also plays an important role in the blood cells’ flow dynamics in both in vivo and in vitro environments. Extensional effects, or a combination of shear and extensional effects, can happen in several situations, such as in micro-contractions (due to velocity transition), in bifurcations (around the apex region and small branch), and when cells flow from a wide blood vessel to a narrow catheter or needles. This latter situation can generate extremely high extensional flows, which can promote hemolysis and, as a result, can lead to clogging and jamming within the devices [28,35]. Hence, recently, several extensional flow studies have been performed not only to assess cell deformability [19,28,29,31,32,33], but also to separate blood cells from plasma [29,30]. The majority of these studies were performed at hyperbolic converging microchannels where single-cell deformability was assessed under a controlled homogeneous extensional flow field. Figure 3 shows RBCs flowing through the expansion region (A) and hyperbolic contraction region (B) for two different flow rates, i.e., 9.45 µL/min and 66.15 µL/min. These qualitative flow visualizations clearly show that the RBC deformability is higher in the hyperbolic contraction region (B) where the RBCs are subjected to a strong extensional flow. Right after the exit of the contraction, RBCs tend to recover their initial shape (A), which corresponds to a minimal value of the deformation and where the RBCs are no longer exposed to a strong extensional flow. Another expected result is that the RBCs have a tendency to increase the deformation as the flow rate increases. More detailed information can be found in the work performed by Yagimuma et al. [29] where they have investigated the influence of the extensional flow on the motion and deformability of individual RBCs in the full length of a hyperbolic microchannel.

**Figure 4:**
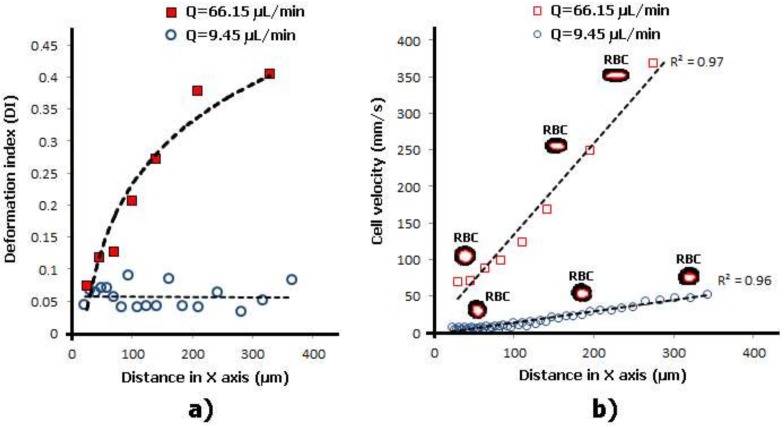
Individual RBCs’ (**a**) DI and (**b**) velocity flowing through a hyperbolic contraction microchannel for two different flow rates: 9.45 µL/min and 66.15 µL/min.

Figure 4 shows a quantitative description of the degree of deformation of human RBCs under a homogenous extensional flow field. We have measured the DI, as well as the velocity of the RBCs flowing through the expansion and hyperbolic contraction regions, for two different flow rates. For an inlet flow rate of 9.45 µL/min the RBCs do not suffer any significant deformation and the DI values are fairly constant along the full length of the microchannel. However, for a flow rate of 66.15 µL/min it is clear that when the RBCs enter the contraction region, RBCs start to elongate and, consequently, their DI values start to increase until the end of the hyperbolic contraction region. The latter results clearly show that when the RBCs are subjected to strong extensional flows RBCs tend to elongate up to a maximum value. Another interesting result shown in Figure 4b is that when RBCs reach the hyperbolic contraction region their velocities increase almost linearly, which corresponds to a constant strain rate. This phenomenon happens for the both tested flow rates. These in vitro blood experiments show the potential of using hyperbolic-shaped microchannels to precisely control and assess changes in RBC deformability in physiological and pathological situations. However, the selection of the geometry and the identification of the most suitable region to evaluate the changes on the RBC deformability under strong extensional flows are crucial and further studies need to be performed in more detail in the near future.

Lee and his collaborators [28] have compared the deformability response of the RBCs to simple shear and extensional flows. Their results have shown that extensional flows generate higher RBC deformability than simple shear flows. Recently, Faustino and her colleagues [33,38] have performed RBC deformation measurements in a hyperbolic-shaped contraction with a low aspect ratio (AR), where RBCs were submitted to both extensional and shear flow. By comparing the DI results performed by Faustino et al. [33,38] with the results obtained with extensional flows [29,40] it is clear that the combination of both extensional and shear flow promote higher RBC deformability. Although hyperbolic converging microchannels with low AR show the most suitable approach to assess the clinical meaning of RBC deformability, further studies should be performed with different flow rates and microchannel dimensions.

### 2.2. Deformability in Smooth and Sudden Contractions

**Figure 5:**
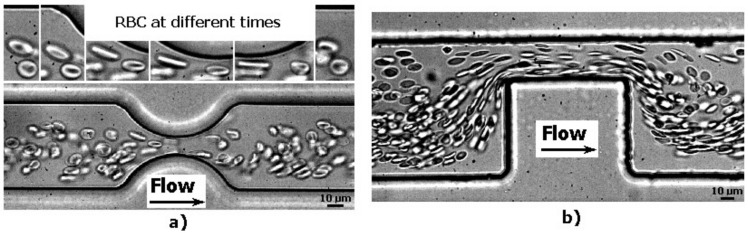
RBCs flowing through a microchannel with (**a**) a smooth and (**b**) a sudden (or abrupt) contraction (adapted from [65]).

During the years, a large number of studies on in vitro blood rheology and particularly in the deformation of RBCs under a simple shear flow were performed by using rotational rheometers [4,19,41,42,43]. However, RBCs flowing in microvessels, due to the confined microenvironment, deform not only due to shear effect but also to extensional effect. Hence, from the beginning of the 21st century, and due to the progress in microfabrication [6,7,44,45], microflow visualization techniques [46,47,48,49,50,51,52,53,54,55,56,57], and image analysis methods [58,59,60,61,62,63], several microfluidic devices containing microchannels have been proposed to study RBC deformability in environments closer to in vivo microcirculation. Most of the proposed microfluidic devices to perform RBC deformability characterization can be classified as fluid-induced deformation microchannels (when the dimensions of the channels used to generate deformability are larger than the tested cells) and as structure-induced deformation microchannels (constriction channels with dimensions similar or smaller than the diameter of tested cells). To the best of our knowledge, the first application of a microfluidic constriction channel to perform RBC deformability measurements was done by Tsukada et al. [20]. In this study they measured RBC deformability of diabetic RBCs flowing through constriction microchannels and they reported that the deformability of diabetic RBCs was lower than healthy RBCs. A few years later, Shelby et al. [21] used a polydimethylsiloxane constriction microchannel to investigate the deformability changes between malaria infected RBCs and healthy RBCs. As expected, they have confirmed that the deformation of the infected RBCs decreases as the parasite progresses. After these two deformability research studies several microfluidic devices, having constriction microchannels, were proposed to measure the deformation of RBCs [5,8,19,26,28,29,32,33,64,65], white blood cells (WBCs) [31,35,66], and cancer cells [22,37]. Although, the majority of the proposed microfluidic devices to perform RBC deformability characterization have focused on the strong shear effects created by the walls, these kinds of devices, due to the extremely small dimensions of the microchannels, have several critical difficulties, including fabrication complexity, flow control, and microflow visualizations. One way to overcome such experimental difficulties is by using fluid-induced deformation microfluidic devices. These kinds of devices are easier to fabricate [6] and, most of the time, produce a combination of shear and extensional flows. Some successful examples, by using abrupt or sudden contractions, are the studies performed by Zhao et al. [25], Forsyth et al. [67], and Fujiwara et al. [68]. Zhao et al. [69] have performed measurements of the RBCs’ deformation in a sudden-contraction microchannel and they have reported that under different flow rates, RBC elongation reached a maximum value and could not deform any further. Forsyth et al. [67], by using a microfluidic constriction channel, have studied the deformability and dynamic behavior of both healthy and hardened RBCs and they have found different types of flow motion due to the increased shear rate in the constriction microchannel. The effect of RBCs deformability on the cell-free layer (CFL) thickness, by hardening RBCs, was also investigated at an abrupt microfluidic constriction channel by Fujiwara et al. [68]. They have found that the RBC deformability plays an important role on the asymmetry of the CFL thickness and they have reported that the motions of RBCs are strongly affected by the deformability, haematocrit, and the channel geometry. However, abrupt constriction microchannels fail to produce homogeneous extensional flows and, as a result, several researchers have been assessing RBC deformability using hyperbolic converging microchannels [19,28,29,30,31,32,33,34]. RBC deformability changes in response to shear and extensional flows strongly depend on the geometric configuration and dimensions of the constriction. For instance, the motion and deformation of a RBC passing through a sudden constriction is different from a RBC passing through a smooth or hyperbolic constriction. Pinho et al. [65] have developed a partial cell separation microfluidic device, where RBC deformability was assessed in different kinds of constriction channels. Figure 5 shows RBCs flowing through a smooth and a sudden (or abrupt) constriction microchannel.

**Figure 6:**
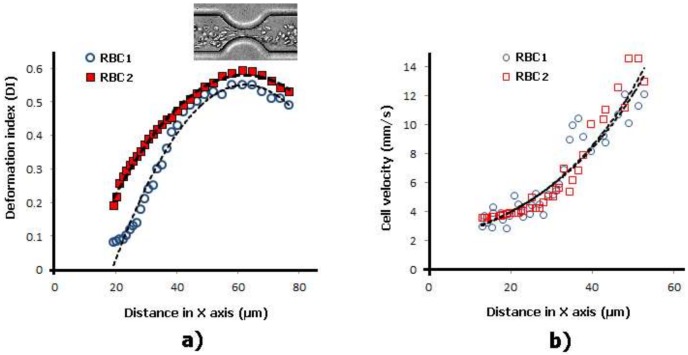
Individual RBCs (**a**) DI and (**b**) velocity flowing through a smooth contraction microchannel for the same flow rate. The X axis correspond to the main flow direction.

**Figure 7:**
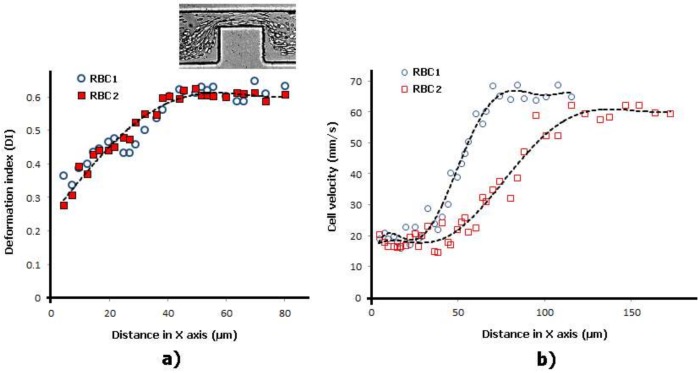
Individual RBCs (**a**) DI and (**b**) velocity flowing through a sudden contraction microchannel for the same flow rate. The X axis correspond to the main flow direction.

In Figure 6 and Figure 7 it is possible to observe the DIs and velocities of two individual RBCs flowing through a smooth and a sudden constriction microchannel, respectively. These results show that for both situations when the RBCs start to enter the constriction region the cells velocities increase and, consequently, they deform up to a maximum value. The measurements performed in a sudden contraction (see Figure 7) show that the RBCs’ elongation tends to reach to a maximum value and, afterwards, do not deform any further due to the constant velocity that cells possess when they flow within the contraction. These latter results are in accordance with the findings performed by Zhao et al. [25]. However, recent results performed by Zeng and Ristenpart [69] have shown that the deformability of the RBCs tend to decrease slightly as they progress within the contraction region. Hence, these contradictory results show that there is a need for further research in this field. However, it is clear that the RBCs flowing through this kind of contraction are not subjected to constant strain rates. This is in contrast to the flow phenomenon that happens in hyperbolic contractions.

It is known that RBCs’ rigidity has been correlated with malaria, sickle cell disease, diabetes mellitus, and others haematological disorders and diseases that affect RBC deformability [1,2,4]. Therefore, several flow studies with rigid RBCs [19,67], or with microparticles that simulate rigid RBCs, have been investigated due to the important role that they play in clarifying the hemodynamic behavior of diseased cells in microcirculation. Pinho et al. [70] have performed a study in order to clarify the flow behavior of both healthy RBCs and rigid microparticles when subjected to high shear rates. In this study, they have investigated the trajectories and DI in a microchannel with a pronounced microstenosis (75%). By using a microfluidic device fabricated by a soft lithography technique, they have used a solution of Dextran 40 containing a mixture of 0.5% polystyrene (PS) latex microspheres (10 µm), that mimic rigid RBCs (arRBCs) mixed with 1% of healthy ovine RBCs (diameter: ~5 µm). The in vitro experiments were performed under different flow rates (1, 10, 20 µL/min) and the DI of both arRBCs and healthy RBCs were measured and compared. More detailed information about the experimental setup can be found elsewhere [70].

**Figure 8:**
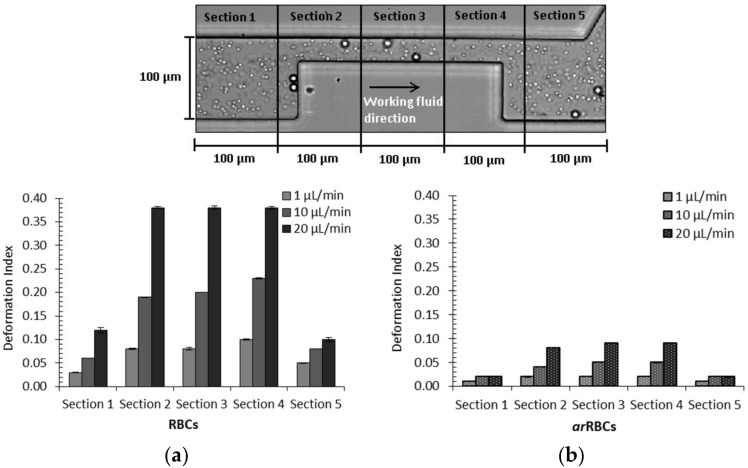
DI measured at five different sections of the stenosed microchannel for different flow rates: (**a**) healthy ovine RBCs; and (**b**) particles mimicking rigid RBCs (arRBCs). Error bars represent a 95% confidence interval (adapted from [70]).

In Figure 8 it is shown that, for both RBCs (rigid and healthy), the maximum DI was obtained at the highest flow rate used in this study and within the stenosis region (represented by Section 2, Section 3 and Section 4). As expected, healthy RBCs had higher DIs when compared with rigid microparticles (arRBCs). In addition, it was at the highest flow rate of 20 µL/min that healthy RBCs obtaining a maximum DI of 0.38 in comparison to the 0.09 obtained by the arRBCs. These results are consistent with the ones obtained by Pinho et al. [65], where healthy human RBCs were investigated using different kinds of constrictions. Additionally, in this study, they have observed that some of the ovine RBCs have changed their normal shape to a parachute or umbrella shape when passing through the sudden constriction microchannel. In contrast, the rigid microparticles did not exhibit any noteworthy change from their original shape. Note that the measured residual values of the arRBC DIs were mainly due to image distortions of the high-speed microparticles.

### 2.3. Deformability in Rectangular PDMS Microcapillaries and Micropillars

**Figure 9:**
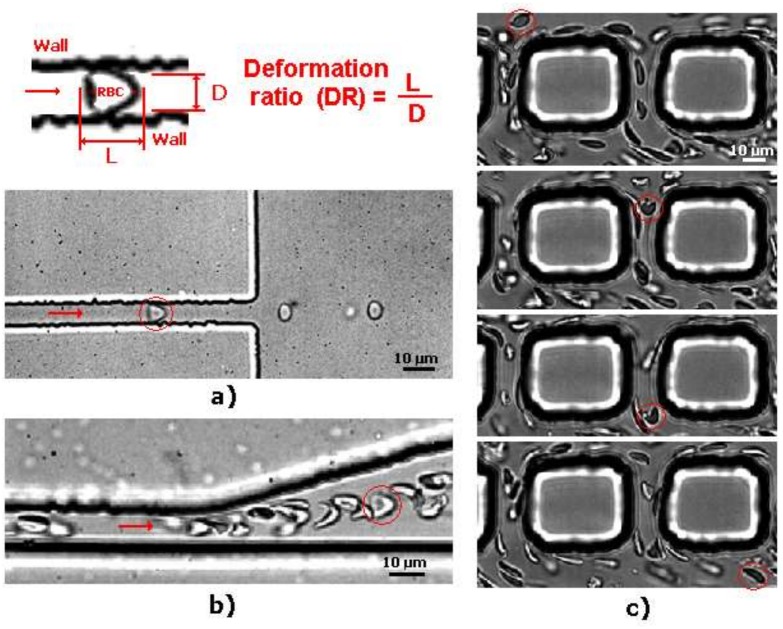
RBCs flowing through (**a**) rectangular PDMS microcapillary (**b**) divergent region upstream of a rectangular PDMS microcapillary; and (**c**) micropillars, adapted from [31].

**Figure 10:**
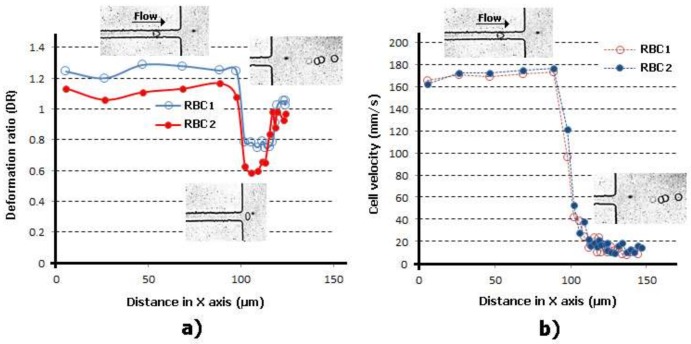
Individual RBCs’ (**a**) DR and (**b**) velocity flowing through a rectangular PDMS microcapillary for the same flow rate. The X axis corresponds to the main flow direction.

Although it is difficult to fabricate and control the flow in constriction microchannels with dimensions similar to RBC diameters, this kind of geometry is one the most popular ways to measure the deformability of RBCs. As it is possible to observe in Figure 9, RBCs flowing through structure-induced deformation microchannels, the RBCs tend to deform into a parachute shape or umbrella shape. Researchers, such as Tsukada et al. [20], Jeong et al. [39], and Tomaiuolo et al. [26], have calculated the RBCs’ deformability by applying the formula L/D, where L and D represent the length and diameter of a deformed RBC, respectively (see Figure 9). Note that, in the present study, this measurement approach is designated as the deformation ratio (DR). By following this approach, we have analyzed and measured the DR of two individual RBCs flowing through a structure-induced deformation microchannel (see Figure 10).

Figure 10 shows the DRs and correspondent velocities of two individual RBCs flowing through a microchannel with dimensions similar to the RBC diameter. The results clearly show an abrupt decrease of both DRs and velocities when the RBCs leave the constriction and enter into an expansion region. It is worth mentioning that as soon as the RBC leaves the constriction region, the RBC changes from a parachute to a nearly circular shape. However, this latter behavior is not always true as it is possible to visualize in Figure 9. In Figure 9a, due to the low local haematocrit and abrupt expansion when the RBC leaves the constriction region, the RBC changes its shape to a circle. In contrast, when the RBC flows within a high local haematocrit and smooth expansion, the RBC tends to keep its parachute shape for a certain period of time. Eventually, when the shear stress induced by the walls decrease the RBC tend to change to a nearly circular shape. Figure 9c shows that, besides the effect of the geometry and local haematocrit, the orientation is also a parameter that plays an important role on the RBC deformability. Although several numerical blood flow studies [71,72,73,74,75,76,77,78] have been proposed to better understand the RBCs’ flow behavior in microchannels and microvessels, our understanding of the RBC motion, orientation, and deformability at the microcirculation level is still far from complete.

### 2.4. Comparison of Cells’ Deformability Studies

Table 2 shows a summary comparing the main features of several cells deformability studies performed in microfluidic devices. Representative features for comparison are the microfluidic technique, blood cell types, main flow phenomenon and the used approach to measure the degree of deformability of the cells.

## 3. Deformation of Bubbles and Droplets 

The deformation of a gas bubble in a simple shear flow depends on the viscosity ratio, λ, and on the capillary number, Ca [79].

The viscosity ratio is defined as:

(1) $$\lambda =\frac{\mu_{g}}{\mu_{l}}$$

where $\mu_{g}$ is the gas viscosity and $\mu_{l}$ the viscosity of the liquid.

The capillary number is defined as:

(2) $$Ca=\frac{W\dot{\gamma }\mu_{l}}{\sigma }$$

where σ is the surface tension, W the characteristic dimension of the flow and $\dot{\gamma }$ the flow shear rate. For Ca≪1 and λ≪1, the deformation is very small and changes linearly with the capillary number.

Müller-Fischer et al. [79] have analyzed the deformation and breakup of bubbles in a parallel band apparatus to understand the influence of the viscosity ratio and the capillary number. The size of the bubbles was approximately 1 mm. The bubbles were subject to deformation under a simple shear flow. By increasing the capillary number, deformation indices of about 0.9 were obtained. As a result, an empirical relation for the deformation index versus the capillary number was obtained and compared with correlations from the literature. Anderl et al. [80] developed a numerical method to predict the deformation of bubbles and were able to predict the results of Müller-Fischer et al. [79]. Wei et al. [81] have used the Lattice Boltzmann method to simulate the deformation of a bubble and were able to correctly predict the shape of the bubble under a simple shear flow. 

Bubble deformation has also been studied in T-junction divergent flows. Fu et al. [82] identified three types of symmetric breakup of bubbles. The first one was controlled by the pressure increase in the liquid phase and the second type was controlled by the pressure increase and viscous effects. In the third type a scaling law for the minimum neck was observed. During the experiments, non-breakup bubbles were observed. As a result, the authors have proposed phase diagrams (capillary number versus normalized bubble length) showing the different conditions to observe various types of bubble behavior. Liu et al. [83] have studied this flow by numerical methods, obtaining detailed velocity and pressure fields, in addition to the bubble shapes and breakup conditions. These authors observed breakup regimes similar to the ones observed by Fu et al. [82].

**Figure 11:**
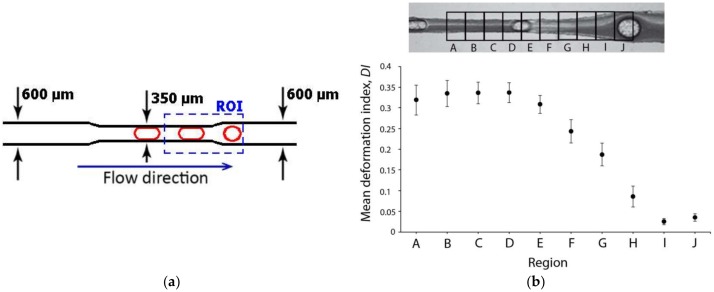
(**a**) Schematic representation of the microchannel contraction region and flow direction of a device to study gas embolisms [85]. The region of interest is indicated by a dotted rectangle; and (**b**) the mean deformation index of bubble flowing through the contraction region.

In microvessels, the flow of air microbubbles may block arterioles and capillaries and, as a result, may stop the supply of blood to certain regions of the human body. Pathological events caused by microbubbles trapped in blood vessels need to be better understood. The shape and velocity of microbubbles in microchannels is known to be dependent on the capillary number and Reynolds number [84]. However, the blood cells present an additional complication. Hence, it is important to improve our understanding of the motion and deformation of microbubbles flowing in microchannels with dimensions similar to in vivo microvessels. A microfluidic system capable of generating air microbubbles was used to investigate the effect of a constriction microchannel on the deformation of individual air microbubbles flowing within in vitro blood. The fabrication technique of a flow-focusing device and the flow conditions of in vitro blood containing microbubbles are presented and discussed in more detail elsewhere [85,86]. Briefly, the microbubbles were produced in the following way: the dispersed phase (air) was squeezed by two counter-streaming blood flows of the carrier phase, forcing the gas to break up and, consequently, the bubbles were generated. Two kinds of bubbles were observed in this microfluidic device: Taylor bubbles and spherical bubbles (Figure 11). The Taylor bubbles were formed and have preserved their shape until they reach the smooth expansion, where they acquired a circular shape. These Taylor bubbles flow through the microfluidic channel separated from each other by liquid slugs and from the wall by a thin liquid film. As expected, the bubbles flowing through the contraction region have higher velocities when compared with the spherical bubbles flowing within the expansion region of the device. Note that the formation of large bubbles in the expansion region of the microchannel was also observed. These latter bubbles are formed mainly due to the collision between them, which led to coalescence. Another point of interest is not only the formation of a cell-free layer around the bubbles, but also the effect of bubbles on the variation of the local hematocrit. This phenomenon is presented and discussed in more detail elsewhere [85].

Figure 11 shows the transition of the DI of a microbubble flowing through the contraction region. Eight individual microbubbles were measured frame by frame and DI values were averaged for 10 different regions (A–J) along the microchannel. Around Region A, the slug bubble is generated. From Region B to D, the DI values are approximately constant, and when the bubble approaches the expansion region it starts decreasing. At Regions I and J, the bubble shapes correspond to an almost perfect circle and tends to keep its shape through this region.

Overall, it is possible to observe certain similar features when the air bubbles and RBCs leave the contraction and enter the expansion region. However, it is also clear that, at the contraction region, the bubbles’ deformability shape significantly differ from the RBCs. For instance, in these experiments the air bubbles do not deform into a parachute shape as it is possible to observe with the RBCs. The capillary number of the experiments is $1.4\times 10^{-4}$. Bubbles in this range of capillary numbers (Ca<0.05) occupy almost all the available channel and do not deform [84].

An overview of the methods used to study bubble and drop deformation is presented in Table 3. Some techniques usually applied to bubbles, such as the imposition of shear flow by moving plates and the use of T-junction divergent flows, have also been applied to drops [87,88]. Hoang et al. [87] have used stop-flow numerical simulations to study the deformation and breakup of droplets in a T-junction divergent flow. They have identified two breakup phases, the first was the deformation dependent on the external flow and, the second, a surface tension-driven rapid pinching leading to breakup. Additionally, drops have been studied in a cross-slot divergent flow in a microfluidic device [89]. The deformation of the drop was dependent on $Ca\cdot \delta^{2}$, where *Ca* is the capillary number and δ is a confinement parameter equal to the ratio between the drop size and the microchannel depth. Hyperbolic contractions have also been used to study not only the deformation of drops in systems with different surfactants [89], but also the deformation and breakup of Pickering droplets [86]. In this configuration the drop deformation depends on the capillary number and on the confinement parameter. 

## 4. Conclusions and Future Directions 

Microfluidic devices have the advantage of being suitable to deal with single-cell deformability while testing large numbers of cells in one single run. This high throughput ability, together with the ability to achieve a controlled flow, make it possible to detect small changes in RBC deformability in a more efficient and less time-consuming way when compared with other deformability measurement techniques, such as micropipette aspiration, rheoscope, and optical tweezers. This review has shown RBC deformability measurements at both fluid- and structure-induced deformation microfluidic devices. Hence, visualizations and measurements of the deformation of RBCs flowing through hyperbolic, smooth, and sudden-contraction microchannels were investigated and compared. Our comparative results show that RBCs flowing through a hyperbolic contraction experience a strong extensional flow with a region of homogeneous strain rate along the centerline. Hence, hyperbolic-shaped microchannels have shown the potential to precisely control and detect small changes in RBC deformability in pathological situations. A recent haemocompatibility study of RBCs in contact with nanoparticles, has shown that these kinds of microfluidic devices were able to detect small changes of RBC deformability where traditional biocompatibility tests did not show any influence [32]. In conclusion, the hyperbolic-shaped constriction microchannels could be a promising tool to perform sensitive cell deformability measurements and, consequently, to be used as a clinical tool for early detection and diagnosis of blood diseases. However, this technique still facing many challenges, such as the use of low-cost micro-visualization equipment to quantitatively measure the RBC deformability and the development of fast and reliable image analysis methods able to measure both RBC motion and deformation in an automatic manner.

PDMS microfluidic devices have also proved to be an extremely powerful method to better understand the effect of the flowing air microbubbles on several blood flow phenomena happening at the micro-scale level. Our flow measurements and visualizations have shown that the microbubbles promote the formation of a cell-free layer around it and, as a consequence, the local haematocrit was affected. In the near future we plan to compare the obtained experimental in vitro results with multi-phase numerical models to better understand the effect of the air microbubbles on the blood flow behavior in microchannels and microvessels.

## Figures and Tables

**Table 1:** Techniques to measure RBC deformability under different diseased conditions.

Measurement Technique	Human Diseases	Main Key Features	References
Micropipette aspiration	Sickle cell anemia, malaria	Enables accurate mechanical response of single RBCs, labor-intensive, time-consuming, and involves a typically difficult process of manipulation.	[4,10,11,12]
Optical tweezers	Malaria, sickle cell anemia, diabetes mellitus	Ability to obtain a mechanical response of single RBCs down to the piconewton level; labor-intensive, time-consuming and special human technical skills are required.	[4,13,14]
Atomic force microscopy	Cancer, spherocytosis, thalassemia, diabetes mellitus, sickle cell anemia	Ability to apply forces to RBC surfaces at the nanoscale level; labor-intensive; time-consuming, and requires expensive equipment.	[4,15,16,17]
Microfluidic ektacytometer	Diabetes mellitus	Homogenous flow, ability to differentiate healthy and diseased cells, labor-intensive and time-consuming process. It is required to label the RBCs to identify them. This latter process may change the RBCs‘ mechanical properties.	[18,19]
Microfluidic constriction channel	Diabetes mellitus, malaria, cancer, abdominal obesity and metabolic syndrome	Reduced space, homogenous flow, label-free, ability to measure a large amount of cells in one single run, potential to precisely control and detect small deformability changes, needs a high-speed video microscopy system combined with an image analysis technique; blockage is likely to happen at constriction microchannels with dimensions similar to the RBC diameter.	[18,19,20,21,22,23,24]

**Table 2:** Comparison of several cells deformability studies performed in microfluidic devices.

Microfluidic Technique	Cell Types	Main Flow Phenomenon	Approach to Measure the Degree of Deformability	Main Advantages	Main Disadvantages	References
Fluid-induced deformation channel	Human RBCs	Poiseuille flow	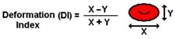	Homogenous flow; ability to measure large amount of cells in one single run.	The extensional flow is not homogenous; expensive micro-visualization equipment.	[25]
Fluid-induced deformation channel	Human&rabbit RBCs, WBCs	Extensional flow (hyperbolic channel)	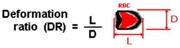	Homogenous extensional flow; high-sensitivity tool; potential to precisely control&detect small deformability changes; ability to measure large amount of cells in one single run.	Expensive micro-visualization equipment.	[19,28,29,32,33,38,40]
Fluid-induced deformation channel	RBCs and WBCs	Extensional flow (cross slot channel)	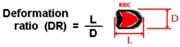	Extensional flow; capacity to differentiate healthy and diseased cells; ability to measure large amount of cells in one single run.	Expensive micro-visualization equipment; the numerical models may need to be validated with in vitro experiments.	[35,36]
Fluid-induced deformation channel	RBCs	Poiseuille flow	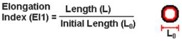	Homogenous flow; ability to measure large amount of cells in one single run.	The extensional flow is not homogenous; expensive micro-visualization equipment.	[67,69]
Structure-induced deformation channel	RBCs	Poiseuille flow	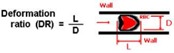	Homogenous flow; ability to differentiate healthy and diseased cells.	Complex to control the flow; difficult fabrication; blockage is likely to happen; expensive micro-visualization equipment.	[20,26]
Structure-induced deformation channel	Cancer cells	Poiseuille flow	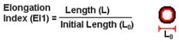	Homogenous flow; ability to differentiate healthy and diseased cells.	Complex to control the flow; difficult fabrication; blockage is likely to happen; expensive micro-visualization equipment.	[22]

**Table 3:** Comparison of several deformability studies performed in microfluidic devices for bubbles and droplets.

Microfluidic Technique	Fluid	Main Flow Phenomenon	Configuration	References
parallel band/plate apparatus	Bubbles and drops	Shear flow	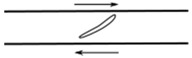	[79,80,88]
T-junction divergence	Bubbles and drops	Extensional flow	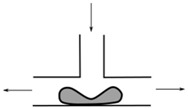	[82,83,87]
Fluid-induced deformation channel	Drops	Extensional flow (hyperbolic channel)	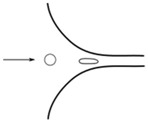	[90,91]
Fluid-induced deformation channel	Drops	Extensional flow	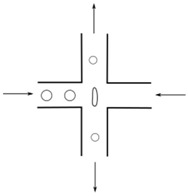	[89]
